# A Novel Adjuvant “Sublancin” Enhances Immune Response in Specific Pathogen-Free Broiler Chickens Inoculated with Newcastle Disease Vaccine

**DOI:** 10.1155/2019/1016567

**Published:** 2019-12-01

**Authors:** Yangke Liu, Jiang Zhang, Shuai Wang, Yong Guo, Tao He, Rui Zhou

**Affiliations:** ^1^State Key Laboratory of Agricultural Microbiology, College of Veterinary Medicine, Huazhong Agricultural University, Wuhan, China; ^2^Linzhou Sinagri Yingtai Biopeptide Co., Ltd, Linzhou, China; ^3^Key Laboratory of Feed Antibiotics Replacement Technology, Ministry of Agriculture and Rural Affairs, Linzhou, China; ^4^National Feed Engineering Technology Research Center, Beijing, China; ^5^Cooperative Innovation Center of Sustainable Pig Production, Wuhan, China; ^6^International Research Center for Animal Diseases (MOST), Wuhan, China

## Abstract

Sublancin is a glycosylated antimicrobial peptide produced by *Bacillus subtilis* 168 possessing antibacterial and immunomodulatory activities. This study was aimed at investigating the effects of sublancin on immune functions and serum antibody titer in specific pathogen-free (SPF) broiler chickens vaccinated with Newcastle disease (ND) vaccine. For this purpose, 3 experiments were performed. Experiment 1: SPF broiler chicks (14 days old) were randomly allotted to 1 of 7 groups including a blank control (BC), vaccine control (VC), and 5 (3-7) vaccinated and sublancin supplemented at 5, 15, 30, 45, and 60 mg activity/L of water, respectively. Vaccinated groups (2-7) were vaccinated with ND vaccine by intranasal and intraocular routes at the 14^th^ day. On 7, 14, 21, and 28 days post vaccination (dpv), the blood samples were collected for the determination of serum hemagglutination inhibition (HI) antibody titer. Experiment 2: SPF broiler chicks were divided into 1 of 3 groups, i.e., blank control (BC), vaccine control (VC), and sublancin treatment (ST). On 7, 14, and 21 dpv, the blood samples were collected for measuring HI antibody titer by micromethod. Experiment 3: the design of this experiment was the same as that of experiment 2. On 7 and 21 dpv, pinocytosis of peritoneal macrophages, B lymphocyte proliferation assay, measurement of CD4^+^ and CD8^+^ T cells, and serum cytokine quantitation were carried out. It was noted that sublancin promoted B lymphocyte proliferation, increased the proportion of CD8^+^ T lymphocyte subpopulations, and enhanced the antibody titer in broiler chickens. In addition, it was also observed that sublancin has the potential to induce the secretion of IFN-*γ*, IL-10, and IL-4. In conclusion, these findings suggested that sublancin could promote both humoral and cellular immune responses and has the potential to be a promising vaccine adjuvant.

## 1. Introduction

Infectious diseases, especially viral diseases, remain one of the most critical challenges in poultry industry partly due to the genetic variation of viruses or the inferior quality of the vaccines. It is widely recognized that the application of vaccines coupled with immunopotentiator could improve the efficacy of vaccination [1]. However, commonly used adjuvants, e.g., aluminum and oil emulsion, are reported to produce some side effects, such as carcinogenesis, strong local stimulation, or failure to enhance immunogenicity of weak antigens [2]. Hence, the development of a new type of adjuvant with low toxicity and high efficiency could be of great significance and of immediate practical value in safeguarding the health-associated risk factors in the poultry industry.

Antimicrobial peptides (AMPs) are various naturally occurring molecules which provide immediate and nonspecific defense against invading pathogens [3]. A number of studies pointed out that AMPs participate in the modulation of the immune response [4, 5]. These immunopotentiating properties of AMPs make them a suitable candidate for the adjuvant design. Sublancin is a 37-amino acid AMP isolated from *Bacillus subtilis* 168 with high stability [6]. In our previous studies, we noted that sublancin alleviated *Clostridium perfringens*-induced necrotic enteritis in broilers mainly by alleviating the inflammatory response [7]. Importantly, we also found that sublancin possess the ability to activate macrophages, thereby protecting mice from cyclophosphamide-induced immunosuppression [8]. In addition, intragastric administration of sublancin induced a mixed immune response of Th1 and Th2 in ovalbumin-immunized mice [9]. These reports elucidated that sublancin could be a promising immunomodulator.

However, the immunomodulatory effects of sublancin on SPF broiler chickens remain poorly understood. Additionally, whether sublancin can improve the immune response of ND vaccine in SPF broilers is yet to be known. Although AMPs can improve the cellular and humoral immunity in animals [4], whether sublancin exhibits similar effects in SPF chickens remains to be investigated. Therefore, the present study evaluated the effects of sublancin on immune response for inducing humoral and cellular immunity against ND vaccine in SPF broilers.

## 2. Material and Methods

All experiments involving animals were approved by the China Agricultural University Institutional Animal Care and Use Committee (ID: SKLAB-B-2010-003).

### 2.1. Preparation of Sublancin

Sublancin was produced in our laboratory using a highly efficient expression system involving *Bacillus subtilis* 800 as described previously [10]. The amino acid sequence of sublancin was determined as GLGKAQCAALWLQCASGGTIGCGGGAVACQNYRQFCR, and the peptide purity was >99.6% as determined by high-performance liquid chromatography. Sublancin was produced as lyophilized powder and stored at –20°C until further use.

### 2.2. Animals

Fourteen-day-old SPF broiler chicks were obtained from the Quality Control Department of Beijing Merial Vital Laboratory Animal Technology Co., Ltd. (Beijing, China) and were housed under standard conditions of temperature (22-26°C), relative humidity (40-65%), and light intensity (150-300 lux). The broilers were fed with Co^60^-irradiated sterile nutritious feed in Complete Feed (Beijing Keao Feed Co., Ltd, Beijing, China) while clean and fresh water was made available *ad libitum*.

### 2.3. Experimental Design

#### 2.3.1. Experiment 1

Ninety-one, 14-day-old SPF broiler chicks were randomly allotted to 1 of 7 groups with 13 chicks in each treatment. The treatments included a blank control (BC), vaccine control (VC), and 5 sublancin treatments in which sublancin was supplemented at 5, 15, 30, 45, and 60 mg activity/L of water, respectively. Briefly, soluble sublancin powder was mixed in 1-L drinking barrel located in each group at the rate of 5, 15, 30, 45, and 60 mg activity/L of water. Fresh sublancin was administered daily throughout the experiment. When the barrel containing sublancin was emptied, purified water without treatment was added to the barrel for the remainder of the day. The broilers in the BC and VC treatments had access to purified water without sublancin treatment all day. All the broilers except the BC group were vaccinated with LaSota ND vaccine by intranasal and intraocular routes at the 14^th^ day. On 7, 14, 21, and 28 dpv, the blood samples were collected from the brachial vein for the determination of serum HI antibody titer by micromethod.

#### 2.3.2. Experiment 2

Thirty, 14-day-old SPF broiler chicks were divided into 1 of 3 groups with 10 chicks in each group. The experimental treatments were similar to Exp. 1 except only one sublancin treatment was used in this experiment. In the ST group, birds were provided purified water mixed with sublancin at 30 mg activity/L of water and vaccinated with ND vaccine as in experiment 1. On 7, 14, and 21 dpv, the blood samples from the brachial vein were collected for the determination of HI antibody titer by micromethod.

#### 2.3.3. Experiment 3

Thirty-six, 14-day-old SPF broiler chicks were randomly allocated to 1 of 3 groups with 12 chicks in each group. The design of this experiment was the same as that of experiment 2. On 7 and 21 dpv, 6 chickens per group were selected randomly for the determination of pinocytosis of peritoneal macrophages, B lymphocyte proliferation assay, measurement of CD4^+^ and CD8^+^ T cells, and serum cytokine quantitation.

### 2.4. Serum HI Antibody Assay

Blood samples (0.5 mL per chick) were collected from the brachial vein, put into 2 mL Eppendorf tubes, and allowed to clot at 37°C for 2 h. Serum was separated by centrifugation at 3000 rpm for 15 min for the determination of HI antibody. Serum HI antibody assay was performed as previously described [11]. The geometric mean titer was presented as reciprocal log_2_ values of the highest dilution that displayed HI.

### 2.5. Determination of Pinocytosis of Peritoneal Macrophages

Peritoneal cells were harvested by peritoneal lavage with 20 mL RPMI-1640 (Gibco) medium. The cell-rich lavage fluid was aspirated and centrifuged at 1500 rpm for 15 min. The pellet was resuspended at 1 × 10^6^ cells/mL in RPMI-1640 medium supplemented with 10% fetal bovine serum (FBS) and 100 units/mL penicillin/streptomycin (Life Technologies) and seeded in 96-well plates at 100 *μ*L/well. Cells were purified by adherence to culture plates for 3 h. Thereafter, the culture medium was discarded and 100 mL/well of 0.075% neutral red was added and incubated for 1 h. After washing with PBS for 3 times, 200 *μ*L of lysis solution (alcohol : acetic acid, 1 : 1 *v*/*v*) was added into each well and maintained at 37°C for 10 min. The absorbance was measured at 570 nm by a microplate reader (IMARK type, Bio-Rad, USA).

### 2.6. Proliferation Assay of B Lymphocyte

Blood samples from the heart were collected and then carefully layered on the surface of the lymphocyte separation medium. After centrifugation at 1500 rpm for 15 min, a white cloud-like lymphocyte band was collected and washed twice with RPMI-1640 medium. The cell pellet was resuspended at 1 × 10^6^ cells/mL with RPMI-1640 medium and seeded in 96-well plates at 80 *μ*L per well, then another 20 *μ*L LPS (10 *μ*g/mL) was added. The plates were incubated at 37°C in a humidified atmosphere with 5% CO_2_. After 44 h, 20 *μ*L of MTT (5 *μ*g/mL) was added into each well. The plates were reincubated for 4 h and then centrifuged at 1500 rpm for 10 min. The supernatant was removed carefully, and 100 *μ*L of DMSO was added into each well. The absorbance at 450 nm was measured by a microplate auto reader as the index of B lymphocyte proliferation.

### 2.7. Measurement of CD4^+^ and CD8^+^ T Cells

Cellular populations in the peripheral blood from the broilers were analyzed using flow cytometry. The lymphocytes were stained with CD3-PE, CD4-FITC, and CD8-SPRD at 4°C for 30 min and then analyzed by flow cytometry (Gallios, Beckman Coulter, Brea, CA, USA). The antibodies were purchased from Southern Biotech.

### 2.8. Serum Cytokine Quantitation

Blood samples from the brachial vein were allowed to clot at 37°C for 2 h and subsequently centrifuged at 3000 rpm for 15 min to separate the serum. The concentrations of INF-*γ*, IL-2, IL-4, and IL-10 in serum were measured using commercially available chicken Enzyme-Linked Immunosorbent Assay (ELISA) kits (Cusabio Biotech Company, Wuhan, China).

### 2.9. Statistical Analysis

All the data were analyzed by ANOVA using SPSS Version 20.0 (SPSS Inc., Chicago, IL). Statistical differences among treatments were determined using Duncan's Multiple Range Test. Results are presented as means ± SD. *P* value < 0.05 was considered significant.

## 3. Results

### 3.1. Experiment 1

#### 3.1.1. The Dynamic Changes of Antibody Titer

**Figure 1:**
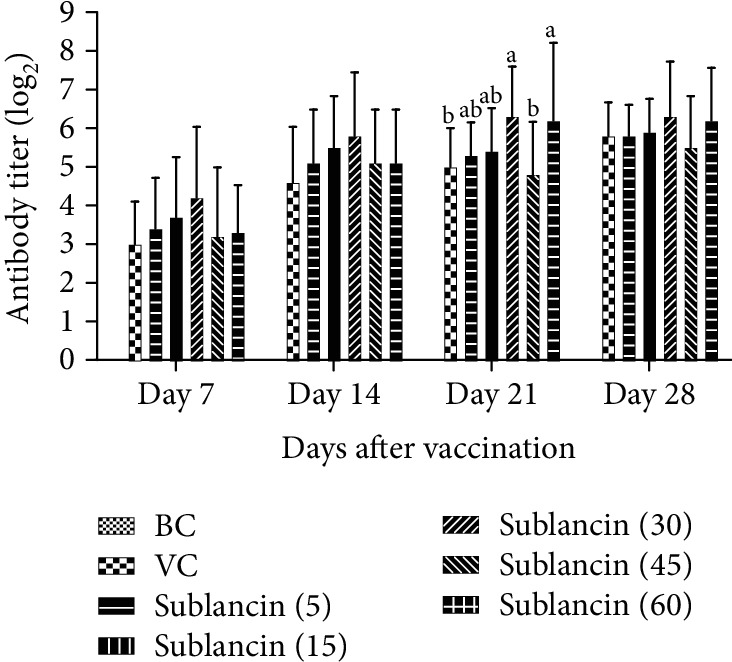
The dynamic variation of HI antibody titer in each group (log_2_) in Exp. 1. ^a,b^Bars in the same day without the same superscripts differ significantly (*P* < 0.05).

The dynamic changes of antibody titer in experiment 1 are presented in Figure 1. On 21 dpv, the sublancin treatments with 30 and 60 mg activity/L of water significantly increased (*P* < 0.05) the antibody titer compared with the VC group. A numerical increase in antibody titer was observed in the 5 sublancin treatments compared with the VC group on 7, 14, and 28 dpv, although there was no statistical difference. Overall, compared with the VC group, the sublancin treatments increased the antibody titer by 1.72~40%.

### 3.2. Experiment 2

#### 3.2.1. Effect of Sublancin on Serum ND Antibody Titers

**Figure 2:**
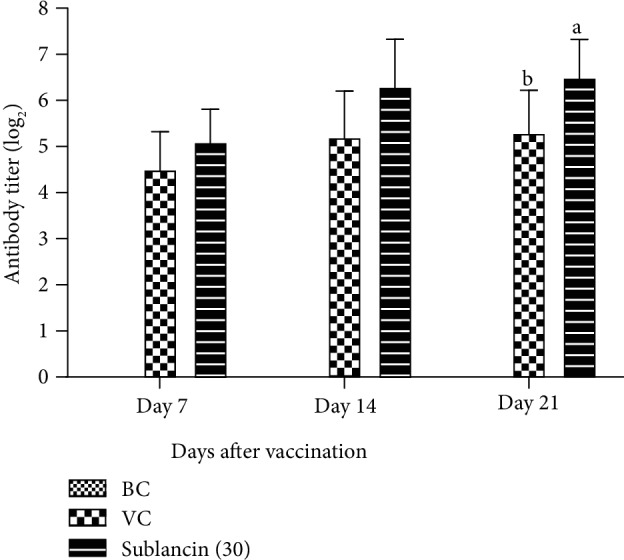
The dynamic changes of antibody titer in each group (log_2_) in Exp. 2. ^a,b^Bars in the same day without the same superscripts differ significantly (*P* < 0.05).

Figure 2 shows the effect of sublancin on serum ND HI antibody titers in experiment 2. In agreement with the results of experiment 1, the antibody titers in the sublancin treatment with 30 mg activity/L of water were significantly higher (*P* < 0.05) than those in the VC group on 21 dpv. On 7 and 14 dpv, the sublancin treatment with 30 mg activity/L of water resulted in a numerical increase in antibody titers by 11.76 and 21.15% compared with the VC group, although there was no statistical difference.

### 3.3. Experiment 3

#### 3.3.1. Effect of Sublancin on Pinocytosis of Peritoneal Macrophages

**Figure 3:**
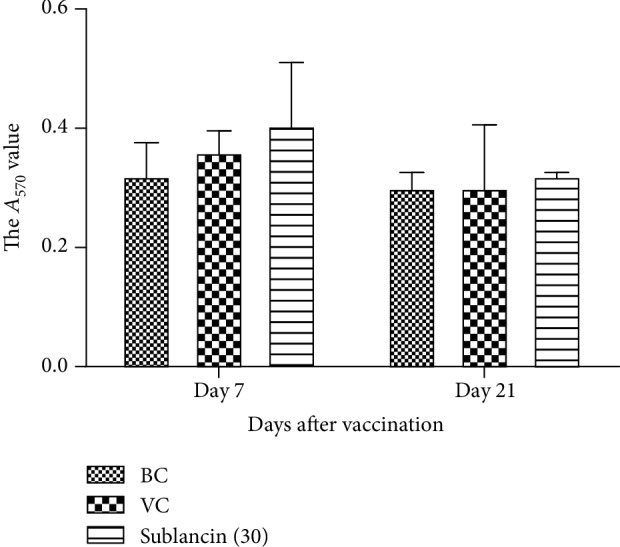
Effect of sublancin on pinocytosis of peritoneal macrophages in Exp. 3.

The pinocytosis activity of broiler peritoneal macrophages was examined by the uptake of neutral red. As shown in Figure 3, the sublancin treatment with 30 mg activity/L of water had no significant effect on the pinocytosis activity compared with the BC and VC groups on 7 and 21 dpv.

#### 3.3.2. The Dynamic Changes of B Lymphocyte Proliferation

**Figure 4:**
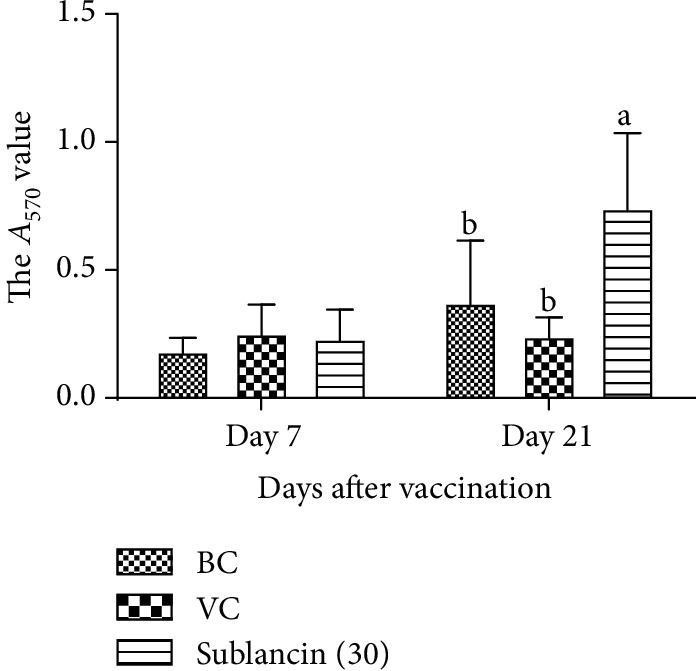
The changes of B lymphocyte proliferation in each group in Exp. 3. ^a,b^Bars in the same day without the same superscripts differ significantly (*P* < 0.05).

The dynamic changes of the *A*_450_ value are presented in Figure 4. On 7 dpv, the *A*_450_ values did not differ among the 3 groups. However, on 21 dpv, the *A*_450_ values in the sublancin treatment with 30 mg activity/L of water were higher than those in the BC and VC groups (*P* < 0.05).

#### 3.3.3. Effect of Sublancin on T Lymphocyte Subpopulations

**Figure 5:**
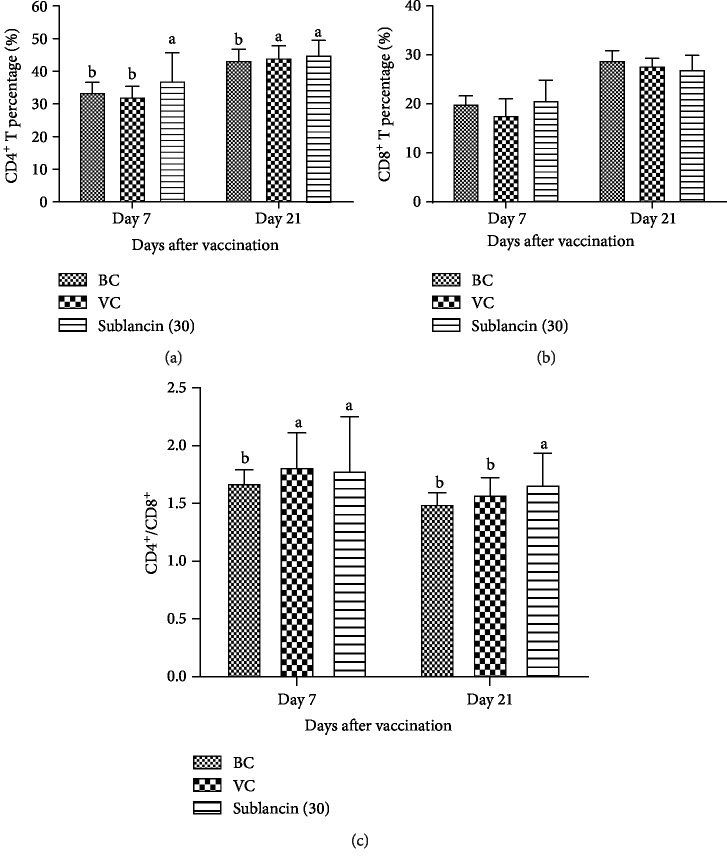
The dynamic changes of CD4^+^, CD8^+^, and CD4^+^/CD8^+^ T lymphocyte subpopulations in each group in Exp. 3. ^a,b^Bars in the same day without the same superscripts differ significantly (*P* < 0.05).

The CD4^+^ and CD8^+^ subsets of T lymphocytes are primarily involved in the immune responses to specific antigenic challenges. We found that the percentage of CD8^+^ peripheral blood lymphocytes in each group remained unchanged between the groups (*P* > 0.05) on 7 and 21 dpv. However, the percentage of CD4^+^ peripheral blood lymphocytes in the sublancin treatment was higher (*P* < 0.05) than that in the BC and VC groups on 7 dpv (Figure 5). Likewise, the values of CD4^+^/CD8^+^ were higher (*P* < 0.05) than those in the BC and VC groups on 21 dpv.

#### 3.3.4. Effect of Sublancin on Cytokine Production

**Figure 6:**
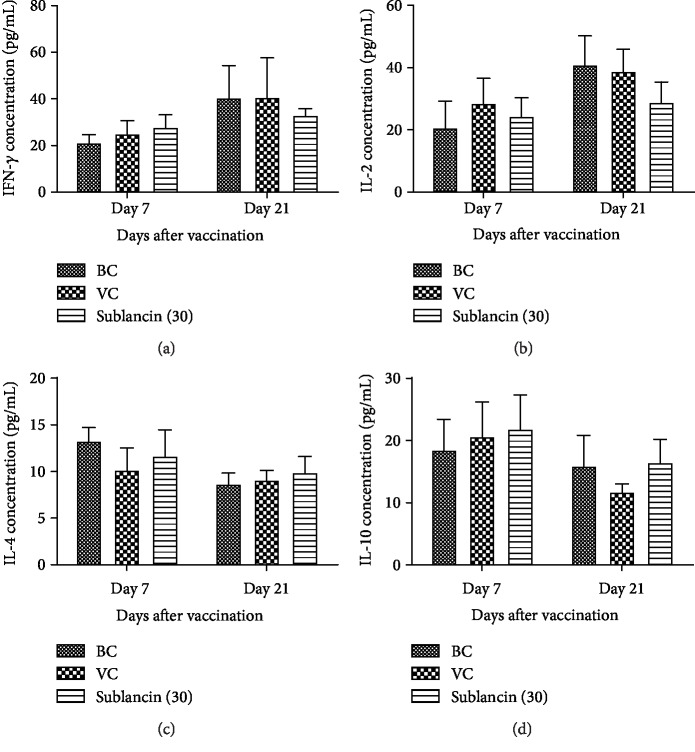
The changes of serum (a) IFN-*γ*, (b) IL-2, (c) IL-4, and (d) IL-10 concentrations in each group in Exp. 3.

As shown in Figure 6, on 7 dpv, a numerical increase in serum concentrations of INF-*γ* and IL-10 was observed in the sublancin treatment compared with the BC group (*P* > 0.05), although there was no statistical difference. On 21 dpv, the IL-4 concentration in the sublancin treatment also showed numerical increase when compared with that in the BC group (*P* > 0.05).

## 4. Discussion

Naturally occurring AMPs exhibit antibacterial properties and are also suggested to possess immune-enhancing activities [12], which make them promising adjuvant candidates for vaccine design. Ribosomally synthesized and posttranslationally modified peptides are a fast-expanding class of natural products that display a wide range of interesting biological activities. Sublancin is a member of the glycocin family containing 2*α*-helices and a well-defined interhelical loop connected by an S-glucosidic linkage to Cys [13]. Mature sublancin has a molecular mass of 3879.8 Da [10]. It has previously been reported that sublancin possesses immunomodulatory properties [8, 14]. Acquired immunity comprising the humoral and cellular immunity constitutes an integral component of bird's health. Humoral immunity mediated by B lymphocytes is a crucial immune reaction against infections, thereby a change in the antibody titer reflects the state of humoral immunity in animals [15]. In our study, sublancin significantly increased the serum ND antibody titers compared with the VC group, suggesting that sublancin could promote humoral immunity.

It is well known that B cells are primarily responsible for humoral immunity, whereas T cells participate in cellular immunity. B lymphocytes mainly secrete antigens by binding the antibodies from effector B cells to eliminate antigens and participate in the humoral immune process of the body. In addition, cytokines can also be released to participate in immune regulation [16]. We noted that sublancin treatment with 30 mg activity/L of water significantly increased the proliferation of B lymphocytes, indicating that B lymphocytes were activated by sublancin. To further test the efficacy of sublancin on cellular immunity, we determined the amount of CD4^+^ and CD8^+^ T lymphocyte subpopulations. CD4^+^ T lymphocytes can be activated by immunoreactive reactions with polypeptide antigens presented by major histocompatibility complex class II molecules. CD8^+^ T lymphocytes recognize antigens presented by major histocompatibility complex class I molecules and directly kill infected or variant cells. The number and status of CD4^+^ and CD8^+^ T lymphocytes directly reflect the status of immunity of the body [17]. Generally, the ratio of CD4^+^/CD8^+^ T remains relatively stable, so the value and proportion of CD4^+^/CD8^+^ T lymphocyte subsets in the peripheral blood and the ability to produce cytokines can be measured in order to assess the immune status of the body cells [18]. Our results showed that the broilers receiving sublancin at 30 mg activity/L of water had an increased value of CD4^+^ T lymphocytes on days 7 and 21 after the vaccination. The value of CD4^+^/CD8^+^ was significantly increased on days 7 and 21 after the vaccination. These results are in agreement with Xiaofei et al. [19] who reported that compound mucosal immune adjuvant can increase the percentage of CD4^+^ T and CD8^+^ T lymphocytes in chicken orally vaccinated with attenuated Newcastle disease vaccine.

Phagocytosis is one of the primary functions of macrophages, and this process is extremely crucial in excluding foreign bodies [20]. In the present study, we evaluated the phagocytic activity of macrophages by phagocytic index *via* neutral red uptake. The results showed that sublancin treatment with 30 mg activity/L of water had no significant effect on the phagocytic activity of macrophages compared with that in the BC and VC groups. These findings suggested that sublancin had no effect on the regulation of macrophages in SPF broilers. On the contrary, our previous study in mice demonstrated that oral administration of sublancin could enhance phagocytic activity of peritoneal macrophages under normal conditions and attenuate the cyclophosphamide-induced inhibition of peritoneal macrophages phagocytic activity [8]. This discrepancy is most likely due to a species difference or physiological state of the birds.

In addition to stimulating the proliferation of immune cells, sublancin has the potential to induce the secretion of IFN-*γ*, IL-10, and IL-4. The Th1 cytokine IFN-*γ* provides protective immunity against intracellular infections by organisms including bacteria, viruses, and protozoa [21] whereas IL-10 and IL-4 participate in the Th2 immune response. In this study, sublancin was administered via oral route, thus the possibility of a loss of glucosylation, reduction of disulfides, and/or attack by endogenous proteases on this peptide during its transit through the intestine cannot be ignored. Such reactions would modify some or all characteristics of the mature sublancin. Therefore, it can be postulated that the observed effects of sublancin in the present study might be due to the action of a partially modified mature sublancin or sublancin-derived peptides.

## 5. Conclusion

In summary, our study demonstrated that sublancin exhibited immunostimulatory properties which effectively activated B lymphocytes, increased the value of CD4^+^/CD8^+^, enhanced the ability to respond to antigens, and consequently increased the serum ND antibody titers in SPF broilers. Hence, the present study suggested that sublancin is a potential candidate to be a vaccine adjuvant.

## Data Availability

The data used to support the findings of this study are available from the corresponding author upon request.
